# Supplementary material for: Effect of Kaolin Clay and ZnO-Nanoparticles on the Radiation Shielding Properties of Epoxy Resin Composites
Source: Polymers (Basel). 2022 Nov 8;14(22):4801. doi: 10.3390/polym14224801 (PMC9698053; doi:10.3390/polym14224801)
Supplement: Supplementary file 1 [file polymers-14-04801-s001.zip › polymers-2000974-supplementary.pdf]

Effect of kaolin clay and ZnO-nanoparticles on the radiation shielding properties of epoxy resin composites

Mahmoud I. Abbas[1], Abdullah H. Alahmadi[2], Mohamed.Elsafi[1,*], S.A. Alqahtani[1], Sabina Yasmin[3], M.I. Sayyed[4], Mona M. Gouda[1], Ahmed M. El-khatib[1]

[1] Physics Department, Faculty of Science, Alexandria University, 21511 Alexandria, Egypt;
[2] Department of Physics, College of Science, University of Hail, P.O.Box 2440, Hail, Saudi Arabia
[2] Department of Physics, Chittagong University of Engineering and Technology, Chattogram, Bangladesh;
[3] Department of Physics, Faculty of Science, Isra University, Amman 11622, Jordan;

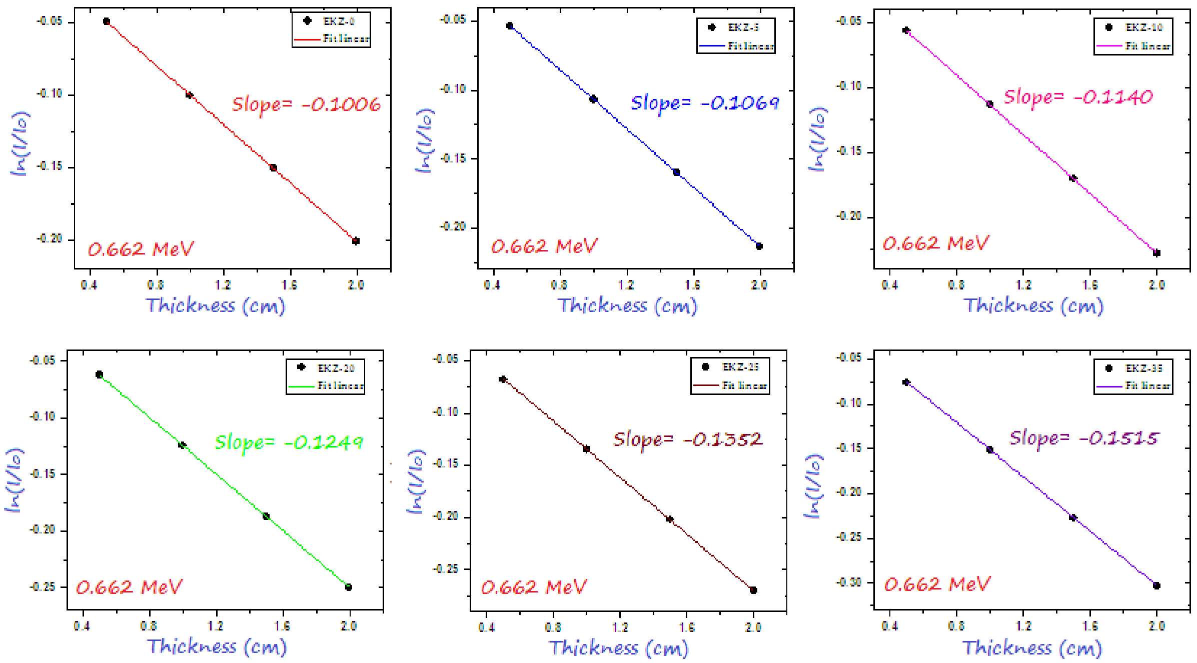

Figure S1. The ln (I/I_0) versus the thickness for all prepared samples at 0.662 MeV.

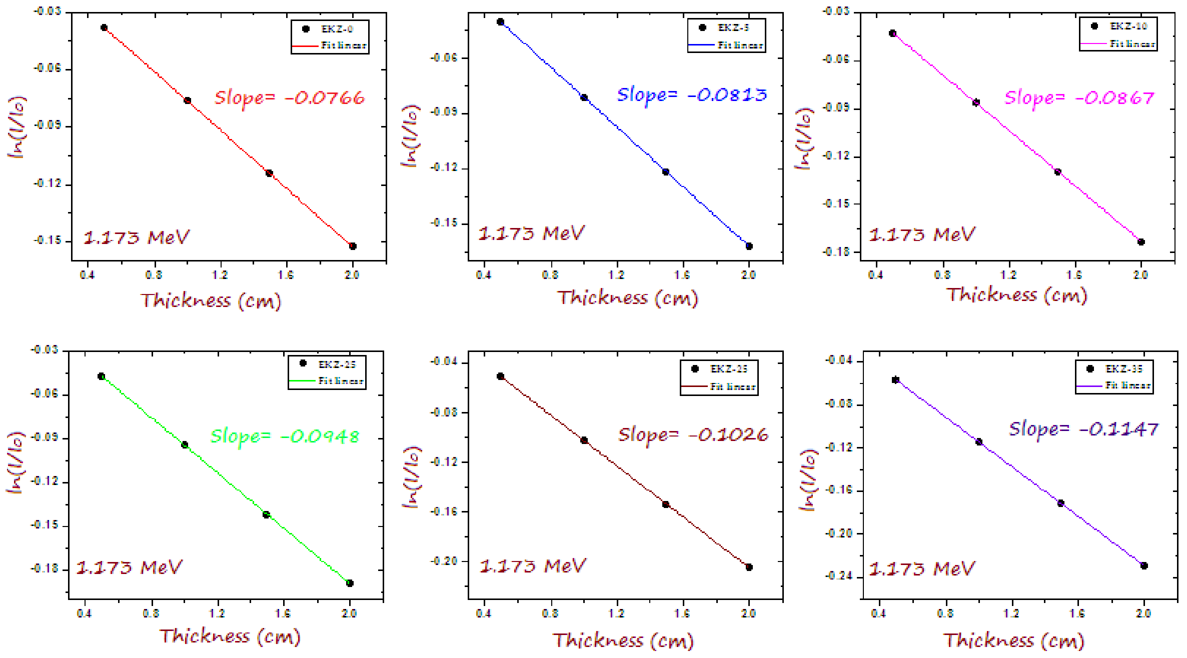

Figure S2. The ln (I/I_0) versus the thickness for all prepared samples at 1.173 MeV.

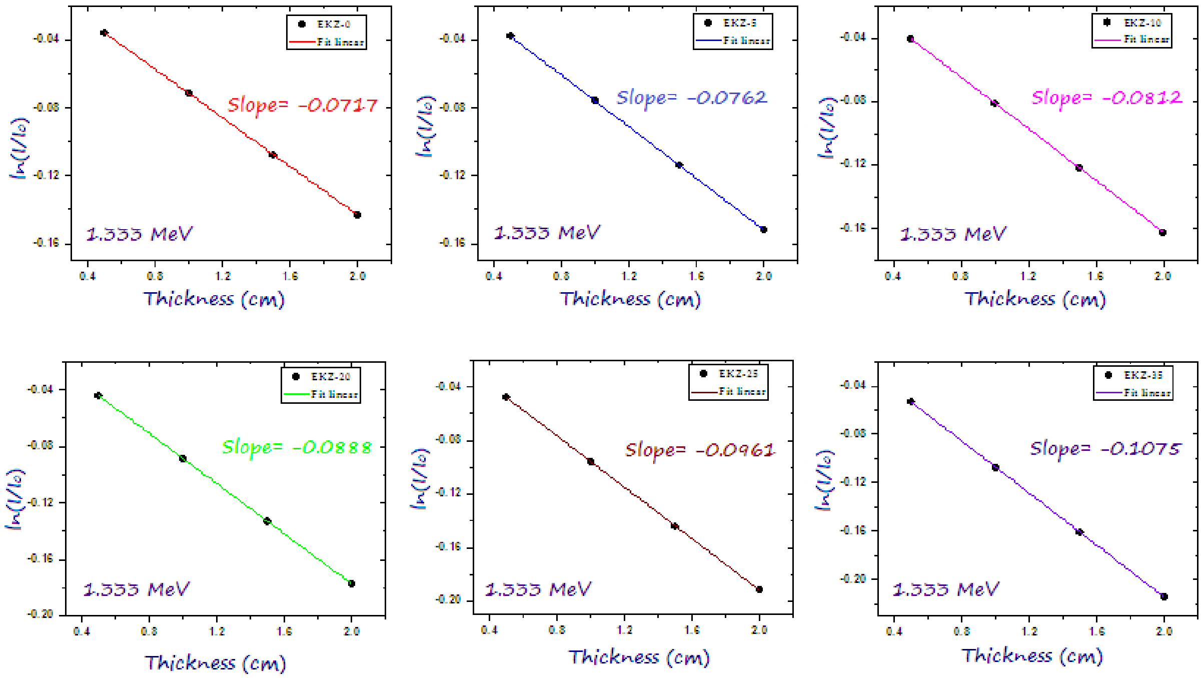

Figure S3. The ln (I/I_0) versus the thickness for all prepared samples at 1.333 MeV.
